# Ripple-Like Descemet Membrane Folds Following Combined Microhook Ab Interno Trabeculotomy and Cataract Surgery: A Case Report

**DOI:** 10.7759/cureus.117708

**Published:** 2026-10-09

**Authors:** Hinako Ohtani, Takayuki Naito, Yoshichika Wanami, Chisako Ida, Takuro Shimoji, Keigo Takagi, Yuto Yoshida, Masaki Tanito

**Affiliations:** 1 Department of Ophthalmology, Shimane University Faculty of Medicine, Izumo, JPN

**Keywords:** cataract surgery, corneal biomechanics, descemet membrane folds, irrigation/aspiration, microhook ab interno trabeculotomy, minimally invasive glaucoma surgery

## Abstract

We report a case of distinctive ripple-like Descemet membrane folds after combined microhook ab interno trabeculotomy (µLOT) and cataract surgery. A 70-year-old Japanese man with primary open-angle glaucoma and cataract underwent nasal µLOT followed by phacoemulsification and intraocular lens implantation in the right eye. At 1 week postoperatively, unusual ripple-like Descemet membrane folds, which had not been noted at the examination on postoperative day 1, were observed in the inferior cornea with localized corneal edema. Best-corrected visual acuity was 0.4 in decimal notation. Multimodal imaging revealed no apparent Descemet membrane detachment or tears. Review of the surgical video showed inadvertent aspiration of the Descemet membrane during irrigation/aspiration (I/A), followed by the appearance of the folds. The patient was managed conservatively, and the folds resolved completely by 3 months postoperatively, with best-corrected visual acuity improving to 1.2. We hypothesize that incision of peripheral supporting structures during µLOT may have reduced tangential tension in the Descemet membrane, increasing its susceptibility to aspiration during subsequent I/A. This mechanism remains unconfirmed. Careful I/A manipulation following µLOT is warranted, and conservative management may be appropriate for similar folds without associated Descemet membrane detachment or tears. As minimally invasive glaucoma surgery is often performed by ophthalmology trainees and surgeons with limited surgical experience, this report may serve as an educational example of an intraoperative complication during combined surgery.

## Introduction

With the growing adoption of minimally invasive glaucoma surgery (MIGS), combined cataract and glaucoma surgery has become increasingly common. Current MIGS procedures are broadly represented by techniques involving implantation of an angle-based device, such as the iStent® (Glaukos, Aliso Viejo, USA) or Hydrus® (Ivantis, Irvine, USA), and goniotomy procedures that incise the trabecular meshwork without leaving an implant in the eye [1-3]. Tanito ab interno microhook trabeculotomy (µLOT) is classified as a goniotomy-based MIGS procedure [4] and is one of the most commonly performed goniotomy procedures in Japan [5]. In µLOT, a microhook is inserted into Schlemm’s canal under gonioscopic visualization and advanced circumferentially to incise the trabecular meshwork and inner wall of Schlemm’s canal adjacent to the iris root [4]. Anatomically, the trabecular meshwork targeted by µLOT lies immediately adjacent to Schwalbe’s line, which marks the peripheral termination of the Descemet membrane, providing a close spatial relationship between the surgical site and the Descemet membrane.

The major postoperative complications of µLOT include transient intraocular pressure (IOP) elevation and hyphema [6,7]. µLOT was originally described as a procedure involving trabeculotomy over approximately 240° of the nasal and temporal angles [8]. However, because nasal trabeculotomy provides an IOP-lowering effect comparable to that of combined nasal and temporal trabeculotomy while being associated with less postoperative hyphema, a more limited trabeculotomy confined to the nasal angle has increasingly been adopted [9]. Descemet membrane folds are a relatively common finding after intraocular surgery, including cataract surgery [10], and can also be observed during the early postoperative period after µLOT. However, they are rarely described as a major postoperative complication in the literature. Consequently, corneal or Descemet membrane-related complications after µLOT have generally been reported only when more substantial abnormalities, such as Descemet membrane detachment or hematoma, occur [11].

Here, we report a case in which unusual, localized ripple-like folds of Descemet membrane developed in the peripheral cornea after combined µLOT and cataract surgery, representing a distinct morphological pattern from typical transient postoperative Descemet membrane folds. Review of the surgical video suggested that inadvertent partial aspiration of Descemet membrane, without an associated tear, during irrigation/aspiration (I/A) in the cataract procedure contributed to the formation of the folds. We hypothesized that reduced tension of the Descemet membrane associated with the preceding µLOT may have predisposed the membrane to this event. Because MIGS procedures may be performed by ophthalmology trainees and surgeons with relatively limited surgical experience, recognition of this possible mechanism may have important educational implications for the safe performance of combined cataract and MIGS procedures. We therefore report this case together with the intraoperative video findings and discuss the possible mechanism underlying this unusual complication.

## Case presentation

A 70-year-old Japanese man had been referred to Shimane University Hospital 1 year earlier for the treatment of infectious keratitis in the left eye. A bacterial corneal ulcer caused by *Staphylococcus *species was diagnosed in that eye and treated with topical antibiotics, which controlled the infection. During treatment, elevated IOP was noted in both eyes: 29 mmHg in the right eye and 23 mmHg in the left eye. Bilateral primary open-angle glaucoma was diagnosed, and topical tafluprost was initiated once daily in both eyes. Subsequently, tafluprost was replaced with a fixed combination of brimonidine and ripasudil administered twice daily in both eyes. Surgery was scheduled for the right eye because of cataract-related visual impairment and persistently elevated IOP. His systemic medical history was unremarkable except for hypertension treated with medication.

Preoperatively, the best-corrected visual acuity (BCVA), expressed in decimal notation, was 0.4 in the right eye with −2.0 diopters (D) of myopic correction, and IOP measured by Goldmann applanation tonometry was 19 mmHg. No abnormalities were observed in the cornea or anterior chamber, whereas anterior polar and nuclear cataracts were present (Figure 1A). Gonioscopy revealed an open angle with age-appropriate pigmentation (Figure 1B). The vertical cup-to-disc ratio was 0.9. The mean deviation (MD) on Humphrey 30-2 visual field testing (Carl Zeiss Meditec, Dublin, USA) was −6.85 dB, with visual field defects corresponding to the optic disc findings. Specular microscopy (EM-3000; Tomey, Nagoya, Japan) revealed a corneal endothelial cell density (CECD) of 3,004 cells/mm² and a central corneal thickness (CCT) of 530 μm (Figure 1C). The patient underwent small-incision cataract surgery combined with µLOT in the right eye, as described in detail below. Postoperatively, topical 1.5% levofloxacin and 0.1% betamethasone phosphate were administered four times daily for 3 weeks, and a fixed combination of brimonidine and ripasudil was continued twice daily. On postoperative day 1, IOP was 11 mmHg, and the intraocular lens (IOL) was well positioned. Mild hyphema, graded as 100 according to the Shimane University postoperative hyphema grading system [6], was observed, but no other abnormalities were documented (Figure 1D).

**Figure 1:**
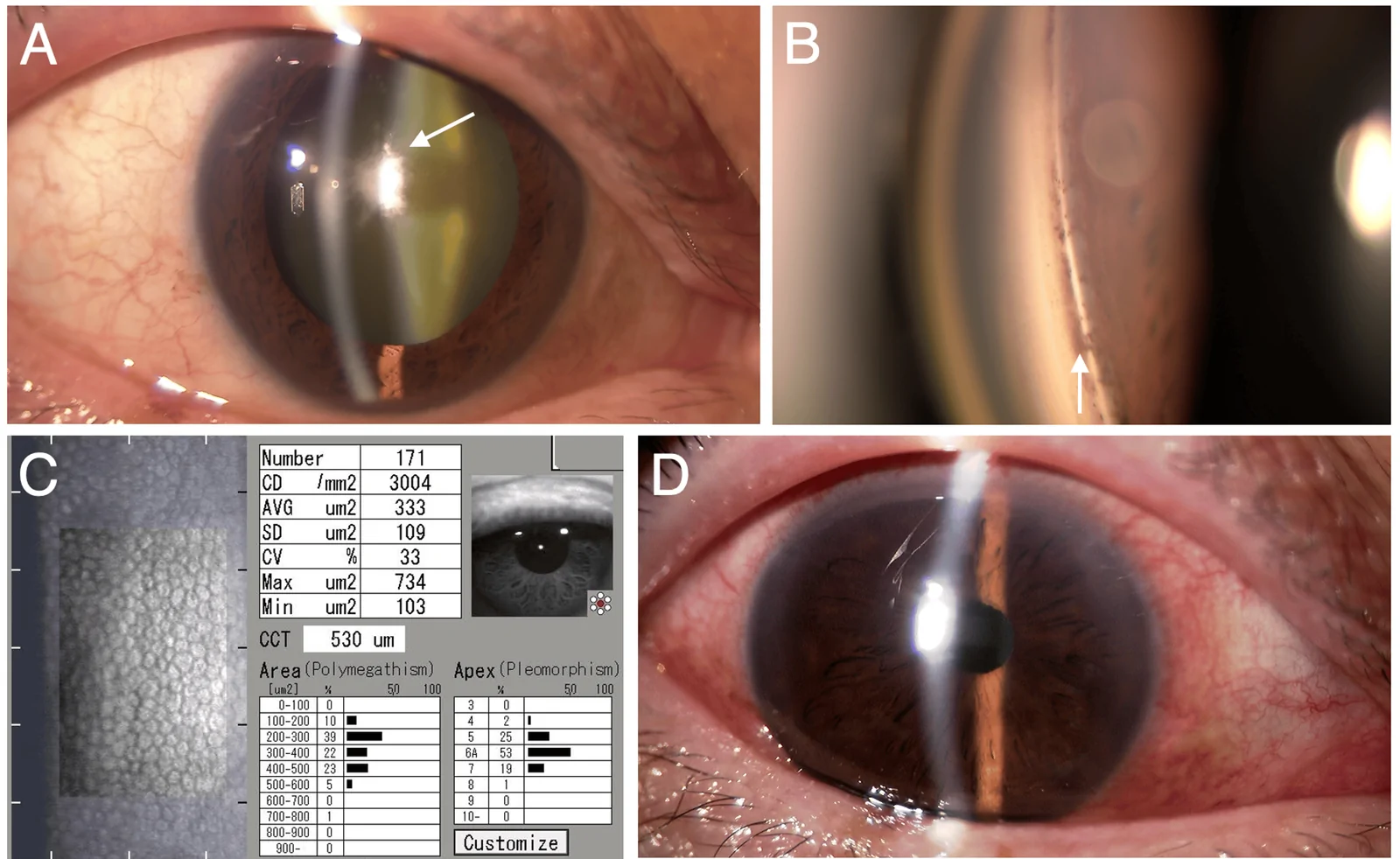
Preoperative (A-C) and postoperative day 1 (D) findings in the right eye. (A) Slit-lamp photograph shows anterior polar (white arrow) and nuclear cataracts. (B) Gonioscopic photograph of the nasal angle shows an open angle with trabecular pigmentation (white arrow). (C) Specular microscopy (EM-3000; Tomey, Nagoya, Japan) shows a corneal endothelial cell density of 3,004 cells/mm² and a central corneal thickness of 530 μm. (D) Slit-lamp photograph shows the findings on postoperative day 1. Intraocular pressure is 11 mmHg. The examining physician does not recognize any corneal abnormalities at this examination. CD, cell density; AVG, mean cell area; SD, standard deviation of cell area; CV, coefficient of variation in cell area; CCT, central corneal thickness.

At 1 week postoperatively, BCVA in the right eye was 0.4, and IOP was 18 mmHg. Unusual ripple-like Descemet membrane folds were observed in the inferior cornea (Figure 2A), without a tear in the Descemet membrane or separation between the Descemet membrane and the corneal stroma (Figure 2B). Anterior segment optical coherence tomography (AS-OCT; CASIA2 Advance; Tomey, Nagoya, Japan) revealed disruption of the concentric corneal thickness pattern inferiorly, suggesting localized inferior corneal edema (Figure 3A). Specular microscopy (EM-3000) showed an increase in CCT to 602 μm compared with the preoperative value. The instrument-reported CECD was 1,262 cells/mm²; however, this measurement was considered unreliable because Descemet membrane folds interfered with accurate endothelial cell analysis (Figure 3B). There was no anterior chamber inflammation or hyphema; the IOL remained well positioned, and no fundus abnormalities were observed. The reduced BCVA was attributed to the Descemet membrane folds and corneal edema, and the patient was observed without changes to the postoperative topical regimen. At 1 month postoperatively, BCVA was 0.6, and IOP was 24 mmHg. A fixed combination of dorzolamide and timolol was added twice daily. Neither AS-OCT (Figure 4A) nor 360° gonioscopic imaging (GS-1; Nidek, Gamagori, Japan; Figure 4B) revealed a clearly identifiable trabeculotomy cleft in the nasal angle.

**Figure 2:**
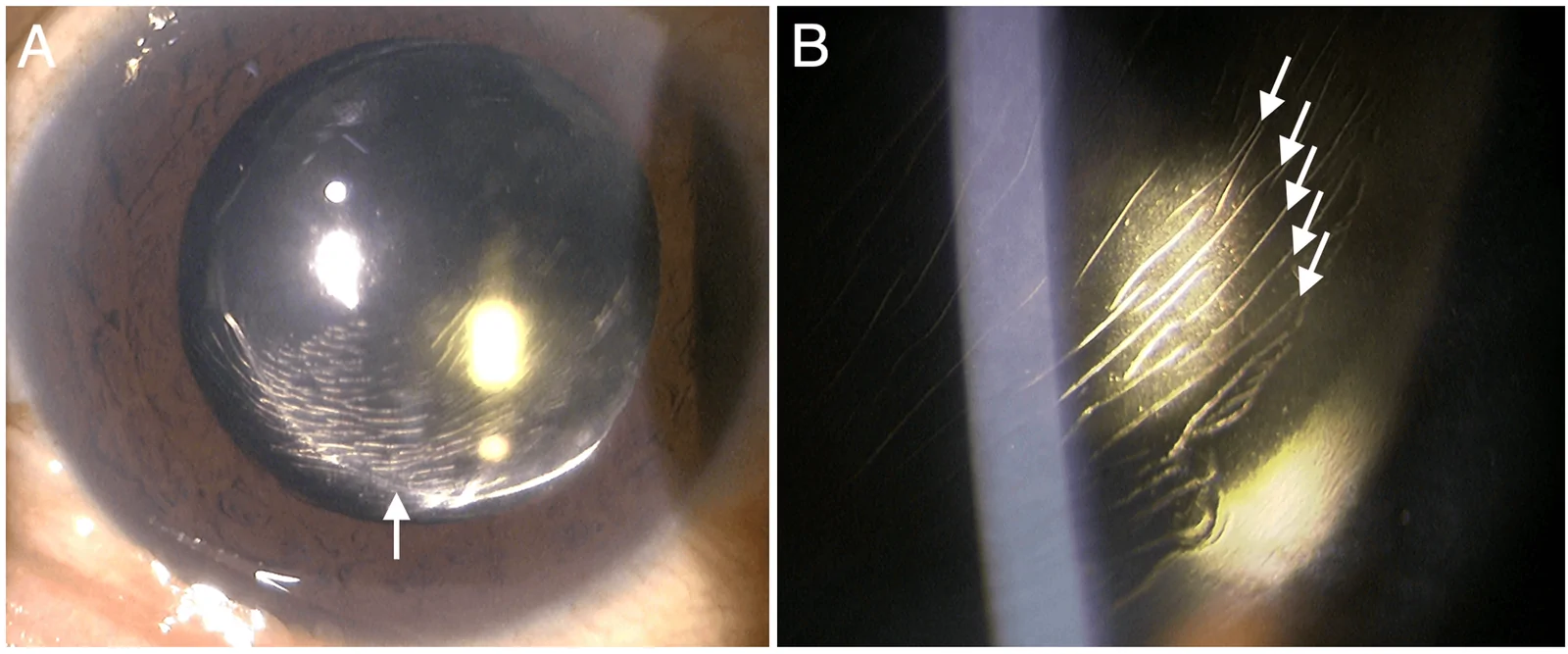
Slit-lamp findings in the right eye one week after surgery. (A) Diffuse illumination shows ripple-like Descemet membrane folds predominantly in the inferior cornea (white arrow). (B) Retroillumination shows Descemet membrane folds without apparent Descemet membrane detachment or marked corneal edema (white arrows).

**Figure 3:**
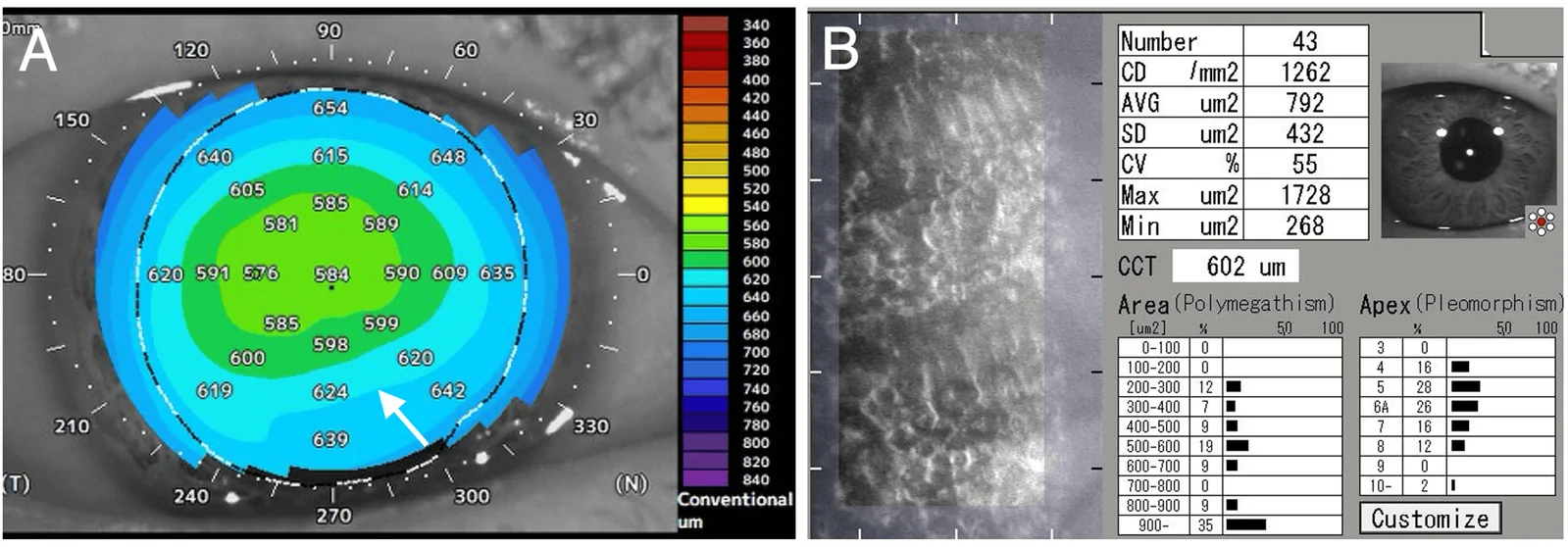
Corneal thickness map (A) and specular microscopy (B) of the right eye one week after surgery. (A) Corneal thickness map obtained using anterior segment optical coherence tomography (CASIA2 Advance; Tomey, Nagoya, Japan) shows disruption of the concentric thickness pattern in the inferior cornea (white arrow). (B) Specular microscopy (EM-3000; Tomey, Nagoya, Japan) shows an increased central corneal thickness of 602 μm. The instrument-reported corneal endothelial cell density is 1,262 cells/mm²; however, this measurement is considered unreliable because Descemet membrane folds interfere with accurate cell analysis. CD, cell density; AVG, mean cell area; SD, standard deviation of cell area; CV, coefficient of variation in cell area; CCT, central corneal thickness.

**Figure 4:**
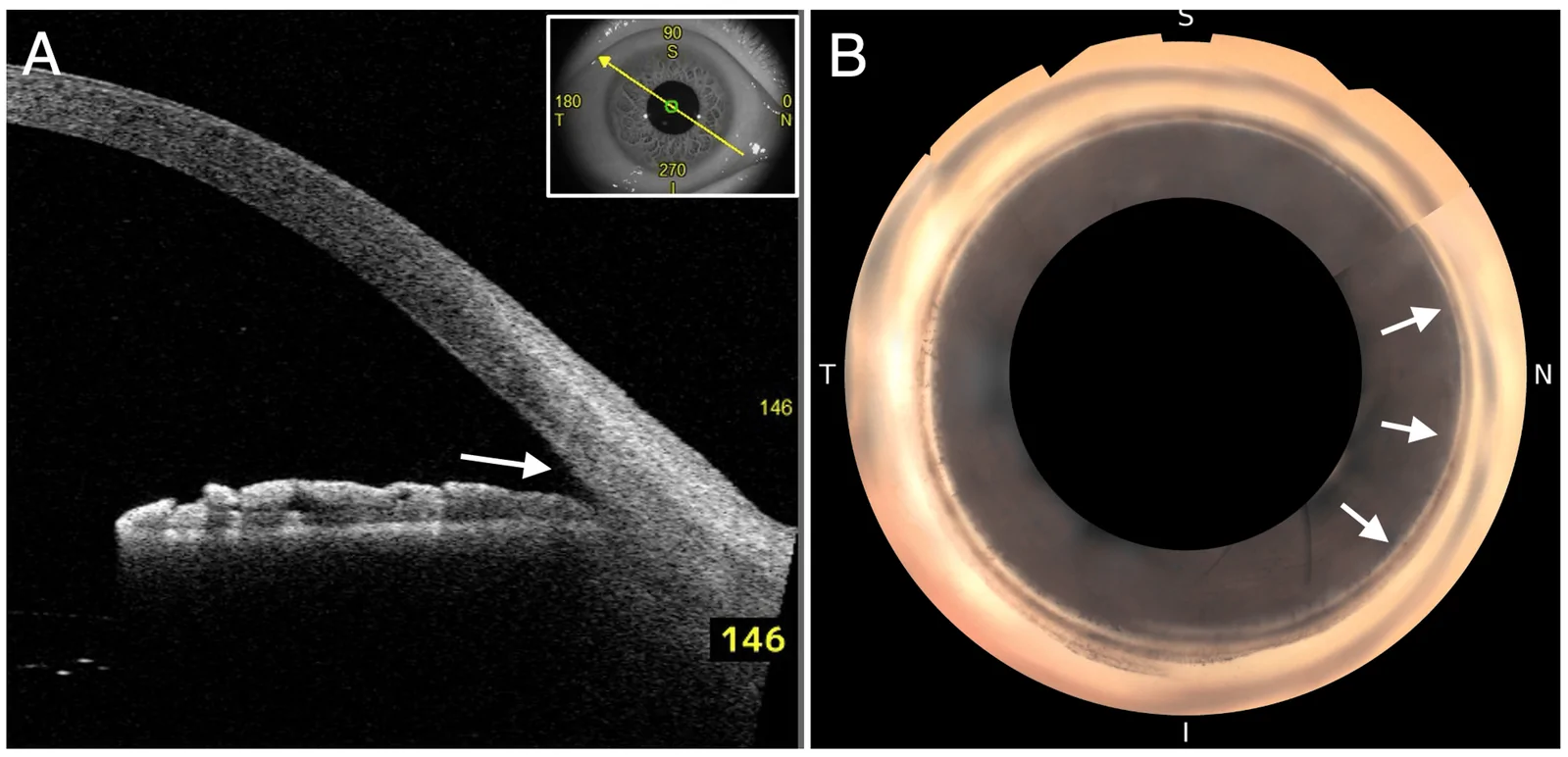
Anterior segment optical coherence tomography (A) and 360-degree gonioscopy (B) of the right eye one month after surgery. (A) Anterior segment optical coherence tomography (CASIA2 Advance; Tomey, Nagoya, Japan) of the nasal angle shows no identifiable trabeculotomy cleft at the indicated site (white arrow). (B) Gonioscopic image obtained using a 360-degree gonioscopy system (GS-1; Nidek, Gamagori, Japan) shows no visible trabeculotomy cleft in the nasal angle (white arrows). T, temporal; S, superior; N, nasal; I, inferior.

The patient continued to be managed conservatively for the corneal findings. At 3 months postoperatively, BCVA in the right eye had improved to 1.2, and IOP was 15 mmHg with twice-daily administration of both fixed-combination brimonidine/ripasudil and fixed-combination dorzolamide/timolol. Slit-lamp examination confirmed complete resolution of the Descemet membrane folds (Figure 5). At the final visit, 6 months postoperatively, BCVA remained 1.2, and IOP was 16 mmHg with the same two fixed-combination eye drops, comprising four active agents. AS-OCT demonstrated restoration of the concentric corneal thickness pattern (Figure 6A). Specular microscopy (EM-4000 Advance; Tomey, Nagoya, Japan) showed a decrease in CCT to 505 μm and a CECD of 2,697 cells/mm². The MD on Humphrey visual field testing was −6.19 dB in the right eye.

**Figure 5:**
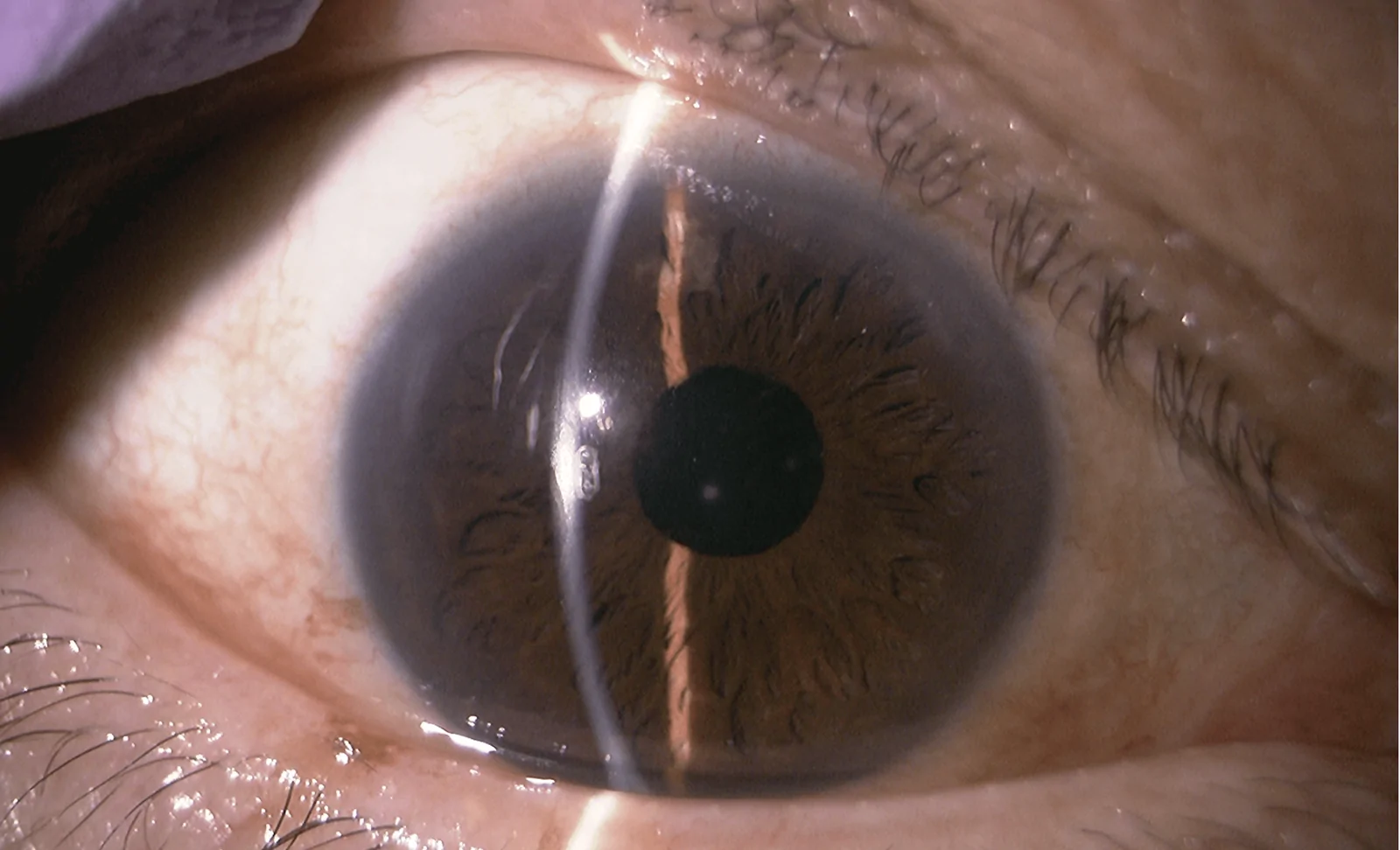
Slit-lamp findings in the right eye three months after surgery. Slit-lamp photograph shows complete resolution of the Descemet membrane folds.

**Figure 6:**
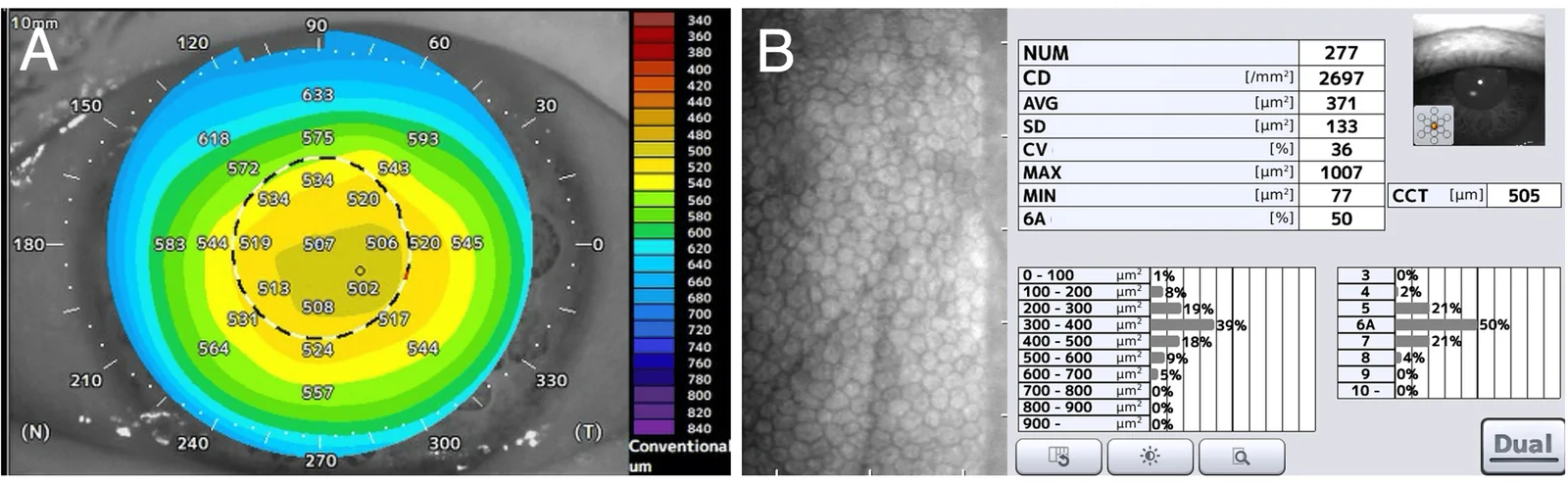
Corneal thickness map (A) and specular microscopy (B) of the right eye six months after surgery. (A) Corneal thickness map obtained using anterior segment optical coherence tomography (CASIA2 Advance; Tomey, Nagoya, Japan) shows restoration of the normal concentric thickness pattern. (B) Specular microscopy (EM-4000 Advance; Tomey, Nagoya, Japan) shows a decrease in central corneal thickness to 505 μm and a corneal endothelial cell density of 2,697 cells/mm². NUM, number of cells analyzed; CD, cell density; AVG, mean cell area; SD, standard deviation of cell area; CV, coefficient of variation in cell area; CCT, central corneal thickness; 6A, percentage of hexagonal cells.

The surgical video was reviewed to investigate the mechanism underlying the formation of the Descemet membrane folds (Figure 7 and Video 1). No abnormalities of the Descemet membrane were evident at the beginning of surgery (Figure 7A). A corneal side port was created nasally at the 10-o’clock position, and the anterior chamber was maintained with ophthalmic viscosurgical devices (ProVisc; Alcon, Fort Worth, USA; and Shellgan; Santen Pharmaceutical, Osaka, Japan). Under visualization with a Swan-Jacob gonioscopic prism (Ocular Instruments, Bellevue, USA), a 90° incision was made in the nasal angle using a Tanito trabeculotomy microhook (Inami, Tokyo, Japan; Figure 7B). The incision line, or trabeculotomy cleft, was located at the junction between the pigmented trabecular meshwork and the scleral spur (Figure 7C). Cataract surgery was then performed through a superior 2.2-mm sclerocorneal incision using the CENTURION system (Alcon, Fort Worth, USA). No corneal abnormalities were evident after phacoemulsification and aspiration of the lens nucleus. During I/A of the residual cortex, however, the Descemet membrane was inadvertently aspirated by the I/A tip, and the Descemet membrane folds became apparent (Figure 7D and Figure 7E). The I/A settings were an irrigation pressure of 50 mmHg, an aspiration flow rate of 30 mL/min, and vacuum set to increase linearly to a maximum of 600 mmHg, consistent with the surgeon’s usual settings. An IOL (+16.5 D, XY-1SP; HOYA Corporation, Tokyo, Japan) was subsequently implanted in the capsular bag. The Descemet membrane folds remained visible during removal of the ophthalmic viscosurgical devices after IOL implantation (Figure 7F). At the end of surgery, a subconjunctival injection of 2 mg betamethasone phosphate was administered, and ofloxacin ophthalmic ointment (Tarivid; Santen Pharmaceutical, Osaka, Japan) was applied.

**Figure 7:**
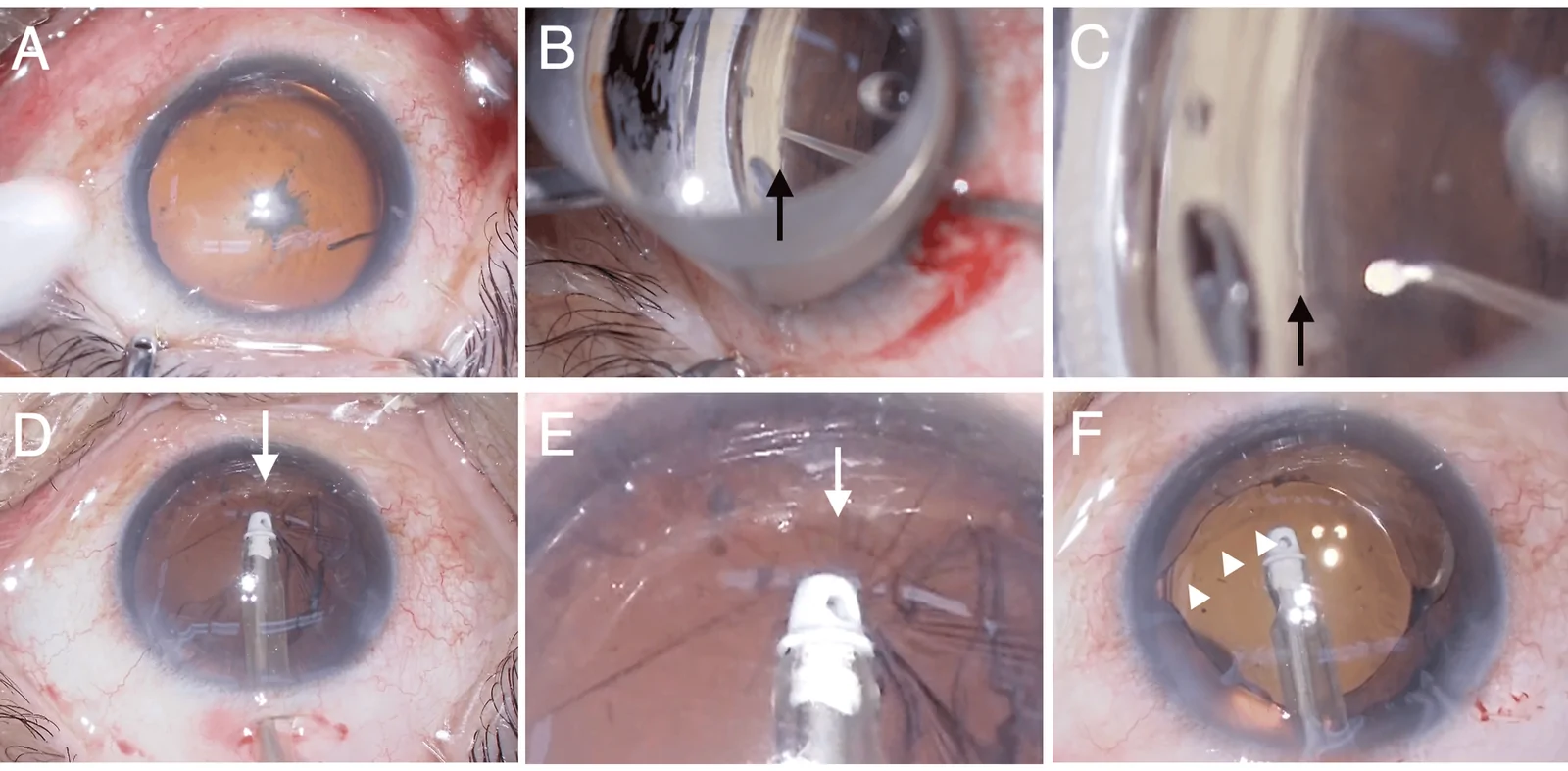
Intraoperative findings in the right eye. (A) Retroillumination at the beginning of surgery shows no apparent corneal abnormalities. (B) The nasal angle is incised using a trabeculotomy microhook under gonioscopic visualization (black arrow). (C) After the angle incision, a linear incision is visible between the posterior border of the pigmented trabecular meshwork and the scleral spur (black arrow). (D, E) During aspiration of residual lens cortex following nucleus removal, Descemet membrane folds are visible as the membrane is drawn toward the irrigation/aspiration tip by suction (white arrows; Video 1, 1:10). (F) Undulating Descemet membrane folds are visible during removal of the residual ophthalmic viscosurgical device after intraocular lens implantation (white arrowheads).

**Video 1:** Surgical video.

## Discussion

We report a case of unusually shaped Descemet membrane folds following combined µLOT and cataract surgery. Multimodal imaging revealed no apparent Descemet membrane detachment or tears. Review of the surgical video suggested that inadvertent aspiration of the Descemet membrane during I/A contributed to the formation of the ripple-like folds. The aspiration appeared to involve a broad area of the membrane. In our clinical experience, this type of aspiration is extremely uncommon during phacoemulsification surgery. In this case, µLOT was performed before cataract surgery, raising the possibility that tissue changes associated with µLOT predisposed the Descemet membrane to inadvertent aspiration during I/A.

Previous studies have reported changes in corneal optical properties and biomechanics following trabeculotomy. A greater extent of Schlemm’s canal incision during goniotomy using µLOT or the Kahook Dual Blade may influence corneal higher-order, coma-like, and spherical-like aberrations after combined trabeculotomy and phacoemulsification [12]. Measurements using the Ocular Response Analyzer and Corvis ST suggested that greater corneal stiffness was a risk factor for higher postoperative IOP measured by Goldmann applanation tonometry after µLOT [13]. Another study reported a greater reduction in corneal stiffness and greater corneal deformation after trabeculotomy than after cataract surgery alone [14]. Significant postoperative changes in Corvis ST parameters, including the stiffness parameter at first applanation and the stress-strain index, have also been observed in both µLOT and control groups, suggesting postoperative corneal softening. These parameters remained unchanged in the iStent group, suggesting that corneal biomechanical changes may differ among procedures involving MIGS combined with cataract surgery, beyond the effects of IOP reduction alone [15]. Collectively, these reports suggest that goniotomy procedures, including µLOT, may alter corneal biomechanics.

Histopathological examination has demonstrated variability in the location and configuration of trabecular meshwork incisions created by µLOT [16]. In a study using intraoperative optical coherence tomography to evaluate trabeculotomy clefts, incision patterns were classified as anterior in 54%, middle in 38%, and posterior in 8% of cases [17]. In the present case, the surgical video showed no obvious incision through the pigmented trabecular meshwork; instead, the incision appeared to involve the trabecular meshwork adjacent to the scleral spur (Video 1, Figure 7C). These findings suggest a posterior incisional pattern. Such incisions may appear as narrow slits, making the trabeculotomy cleft difficult to identify postoperatively. The absence of an identifiable cleft on AS-OCT and 360° gonioscopic imaging in this case was also consistent with this interpretation (Figure 4A and Figure 4B). Pectinate ligaments, also described as ciliary processes or mesodermal remnants, may overlie the trabecular meshwork near the iris root [18]. We hypothesize that incision of peripheral ligamentous structures during µLOT may have reduced tangential tension in the Descemet membrane. This change could have increased the susceptibility of the membrane to aspiration over a broad area during subsequent I/A, potentially contributing to the unusual ripple-like folds observed in this case.

Because no Descemet membrane detachment was present, the patient was managed conservatively without intervention such as intracameral gas injection for membrane reattachment. The folds resolved completely by 3 months postoperatively, and BCVA improved to 1.2. At 6 months, CECD had decreased by approximately 10% from baseline, which was within the range reported after µLOT [7]. IOP was lower than the preoperative value, although this was achieved with an increased number of topical glaucoma medications. The resolution of the folds and improvement in BCVA support the appropriateness of conservative management of the corneal findings in this case.

This report has several limitations. As this is a single case, a causal relationship between µLOT and the development of Descemet membrane folds cannot be established. Although the surgical video suggested that inadvertent aspiration during I/A contributed to fold formation, local tissue deformation and tension were not directly measured. The presumed posterior incisional pattern was inferred from intraoperative findings and postoperative imaging, without histological confirmation of the structures incised. Furthermore, subtle damage to the Descemet membrane cannot be excluded despite the absence of apparent detachment or tears on multimodal imaging. Thus, the proposed mechanism linking incision of peripheral supporting structures to reduced tangential tension and increased susceptibility to aspiration remains hypothetical. Previously reported changes in overall corneal biomechanics do not directly substantiate this local mechanism. Additional cases and experimental studies are needed to evaluate this hypothesis.

## Conclusions

This case highlights inadvertent aspiration during I/A as a potential cause of distinctive ripple-like Descemet membrane folds after combined µLOT and cataract surgery. Reduced membrane tension following µLOT may have predisposed the membrane to aspiration, suggesting a need for careful I/A manipulation and awareness of this possible complication, particularly during surgical training. Complete resolution with conservative management suggests that observation may be appropriate when no associated Descemet membrane tear or detachment is detected.
